# Left Anterior Mini-Thoracotomy *vs.* Conventional Sternotomy in On-Pump Multivessel Coronary Revascularization

**DOI:** 10.21470/1678-9741-2023-0299

**Published:** 2025-05-23

**Authors:** Hüseyin Sicim, Ali Fedakar

**Affiliations:** 1 Department of Cardiovascular Surgery, Kırklareli Training and Research Hospital, Kırklareli, Turkey; 2 Department of Cardiovascular Surgery, Hisar Intercontinental Hospital, Istanbul, Turkey

**Keywords:** Sternotomy, Thoracotomy, Length of Stay, Cardiopulomnary Bypass, Constriction, Drainage

## Abstract

**Objective:**

In this study, we aimed to compare the outcomes of left anterior mini-thoracotomy and conventional sternotomy in on-pump multivessel coronary revascularization.

**Methods:**

Two hundred sixty-two patients who underwent minimally invasive coronary artery bypass grafting through the left anterior mini-thoracotomy and conventional coronary artery bypass grafting with full sternotomy were included. All patients were divided into two groups - 132 patients who underwent minimally invasive multivessel coronary artery bypass grafting in Group I, and 130 patients with full sternotomy in Group II. Intraoperative variables (cross-clamping time, cardiopulmonary bypass time, etc.), postoperative parameters (drainage amount, revision, intensive care and hospital stay times, etc.), and mortality were analyzed retrospectively.

**Results:**

Cardiopulmonary bypass time (152.24 ± 36.4 minutes) was significantly longer in Group I than in Group II (102.24 ± 19.4 minutes) (P<0.001). Cross-clamping time (86 ± 13.2 minutes) was significantly longer in Group I than in Group II (62 ± 21.4 minutes) (P<0.001). And intensive care stay time (P=0.005) and hospital stay time (P=0.004) were significantly shorter in Group I. In the postoperative period, six patients in Group I and seven patients in Group II were revised due to bleeding. Total perioperative mortality was one patient in both groups (P=0.82).

**Conclusion:**

Multivessel coronary artery bypass grafting through the left anterior mini-thoracotomy is an effective, reliable, and successful method, due to less drainage amount and less blood transfusion need, shorter intensive care and hospital stays, faster return to daily life, and better cosmetic results compared to conventional methods.

## INTRODUCTION

**Table:** 

Abbreviations, Acronyms & Symbols	
BMI	= Body mass index
CABG	= Coronary artery bypass grafting
COPD	= Chronic obstructive pulmonary disease
CPB	= Cardiopulmonary bypass
CVA	= Cerebrovascular accident
ES	= Erythrocyte suspension
ICU	= Intensive care unit
LAD	= Left anterior descending
LIMA	= Left internal mammary artery
PAD	= Peripheral artery disease
PDA	= Posterior descending artery
SD	= Standard deviation

Coronary artery revascularization is still the most widely performed cardiac surgery all over the world. Currently, in many centers, total coronary revascularization is still performed with the conventional method by sternotomy. A limited number of minimally invasive approaches are applied to patients with single-vessel or two-vessel disease. Although many centers still continue to apply the conventional sternotomy method in cases with multivessel disease, the popularity of minimally invasive methods in total coronary revascularization continues to increase^[1]^.

Recent studies have shown that this method can be safely applied in a wide spectrum of patients, especially with the left anterior mini-thoracotomy approach^[2]^. Despite the rapid recovery of patients, good cosmetic results, and low cost of this technique, the main limitations are the long learning curve and technical difficulties. However, technological developments and new facilitating techniques are bringing surgeons closer to minimally invasive methods day by day. It is inevitable that minimally invasive methods will become widespread due to the developments in the field of medicine, increasing competition with non-invasive methods in the near future. The important thing is to survive this evolution, which will take place to a certain degree, as successfully as possible. However, the basic principle should be to make the postoperative results as safe and effective as possible.

In this study, we aimed to contribute to the literature by comparing the postoperative outcomes of left anterior mini-thoracotomy and conventional sternotomy in patients who underwent on-pump multivessel coronary revascularization.

## METHODS

From April 2018 to February 2022, a total of 262 patients who underwent minimally invasive on-pump multivessel coronary artery bypass grafting (CABG) through the left anterior mini-thoracotomy and conventional CABG via sternotomy were retrospectively analyzed. The Institutional Ethics Committee’s approval was obtained at 10.02.2022 and numbered 2022/21 project/decision, and written informed consent was obtained from each patient.

All patients were divided into two groups - 132 patients who underwent CABG through the left anterior mini-thoracotomy in Group I, and 130 patients who underwent conventional CABG with classical full sternotomy in Group II. Patients with redo surgery, porcelain aorta, and with concomitant cardiac surgery due to a different concomitant cardiac pathology were not included in the study.

Left internal mammary artery (LIMA) and saphenous vein grafts were used for all anastomoses. Preoperative demographic data of the patients and comorbidities (diabetes mellitus, chronic obstructive pulmonary disease, etc.), intraoperative variables (cardiopulmonary bypass [CPB] time, etc.), postoperative parameters (the amount of transfusion, revision, length of stay in the intensive care unit [ICU] and hospital, etc.), and mortality were analyzed retrospectively.

### Minimal Invasive Surgery Technique

For all patients, Carlens tube is used, allowing single-lung ventilation under general anesthesia. Mini-thoracotomy is performed through a 6-7 cm skin incision from the left side of sternum. A special retractor (Delacroix-Chevalier, Paris, France) is used for LIMA harvesting. For peripheral cannulation, the suitable groin is exposed with a small incision. Femoral artery and vein were cannulated with 20-21F arterial cannula and 24-26F venous cannula. The pericardium was opened, and the distance between the aorta and the pulmonary vein is dissected with help of cautery and encircled with 6 mm tape.

**Fig. 1:**
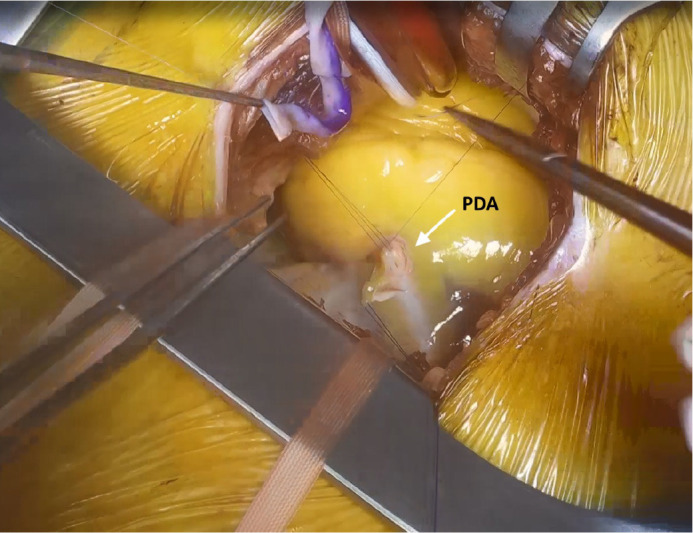
Anastomosis of the saphenous vein graft to the posterior descending artery (PDA).

A cardioplegia cannula is inserted by pulling the aorta with help of the tape. The Chitwood clamp is inserted through the anterior axillary line of the second intercostal space to clamp the aorta. Aorta is cross-clamped, and isothermic blood antegrade intermittent cardioplegia is given. After cardiac arrest is achieved, the pulmonary veins are encircled with 6 mm tape. And then, the inferior vena cava is encircled with 6 mm tape. One side of the saphenous vein graft was marked with methylene blue to avoid twisting. Coronary anastomoses were performed with the standard anastomotic technique of running 7-0 polypropylene sutures (Figure 1).

**Fig. 2:**
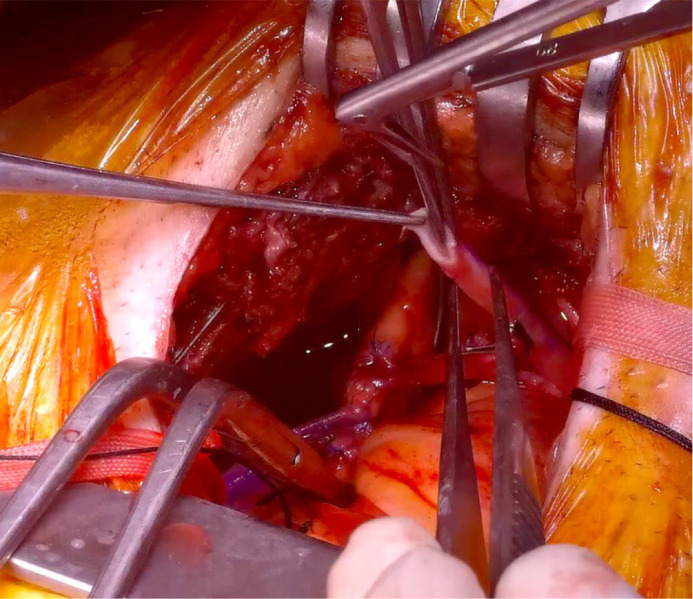
Separate anastomosis of saphenous vein grafts to the proximal aorta.

After the distal anastomoses, a traction is applied to the aorta by placing a side clamp. Standard technique of running 6-0 polypropylene sutures is used (Figure 2).

### Conventional Surgery Technique

Full median sternotomy was performed in the conventional coronary bypass group; after standard anesthesia induction, aortic and atrial cannulation was performed in all patients following the midsternal incision, and CPB was instituted. Aorta was cross-clamped, and isothermic blood antegrade intermittent cardioplegia was given every 20 minutes. Left anterior descending (LAD) artery anastomosis was constructed last. Coronary anastomoses were performed with the standard anastomotic technique of running 7-0 polypropylene sutures. A side-biting clamp is applied on the ascending aorta. Standard technique of running 6-0 polypropylene sutures was used for proximal anastomoses.

### Statistical Analysis

Normal distribution assumption of independent variables was checked by Kolmogorov-Smirnov test. The relationship between the dependent variables and the independent variables were evaluated using the Student’s *t*-test when they fit the normal distribution and the Mann-Whitney U test when they did not fit the normal distribution. Analysis results are considered statistically significant if the confidence interval is 95% and *P*-values are < 0.05. The length of stay in hospital and the ICU was obtained using the Kaplan-Meier method. Analyzes were made with IBM Corp. Released 2016, IBM SPSS Statistics for Windows, version 24.0, Armonk, NY: IBM Corp. package program.

## RESULTS

A total of 262 patients underwent minimally invasive on-pump multivessel CABG through the left anterior mini-thoracotomy and conventional CABG via sternotomy. In Group I, 98 (74.3%) patients were male, and 34 (25.7%) were female, and the average age of the patients was 58.45 ± 8.74 (min-max: 32-78) years; average body mass index (BMI) was 25.26 ± 5.39 (min-max: 19.3-34.5). In Group II, 93 (71.5%) patients were male, and 37 (28.5%) were female, and the average age of the patients was 61.56 ± 19.22 (min-max: 33-80) years; average BMI was 26.2 ± 6.35 (min-max: 20.1-37.2) (Table 1).

**Table 1:** Baseline patients’ characteristics.

	Group I (n=132)	Group II (n=130)	*P*-value
Male:female	98:34:00	93:37:00	> 0.05
Age, years (mean ± SD)	58.45 ± 8.74	61.56 ± 19.22	
Hypertension, n (%)	48 (36%)	52 (40%)	
Diabetes mellitus, n (%)	55 (41%)	49 (37%)	
Hyperlipidemia, n (%)	58 (43%)	51 (39%)	
COPD, n (%)	32 (24%)	28 (21%)	
Smoker, n (%)	45 (34%)	56 (43%)	
PAD, n (%)	3 (2.2%)	5 (3.8%)	
CVA, n (%)	2 (1.5%)	3 (2.3%)	
BMI (mean ± SD)	25.26 ± 5.39	26.2 ± 6.35	
Numbers of anastomosis (mean ± SD)	3.4 ± 0.6	3.6 ± 0.7	

BMI=body mass index; COPD=chronic obstructive pulmonary disease; CVA=cerebrovascular accident; PAD=peripheral artery disease; SD=standard deviation

Average numbers of anastomosis performed were 3.4 ± 0.6 in Group I and 3.6 ± 0.7 in Group II. Average cross-clamping times are 86 ± 13.2 minutes in Group I and 62 ± 21.4 minutes in Group II, and this time was found to be significantly longer in the minimally invasive group (*P*<0.001) (Table 2). In the same direction, CPB duration is 152.24 ± 36.4 minutes in Group I and 102.24 ± 19.4 minutes in Group II. In the comparison between the two groups, this period was found to be significantly longer in the minimally invasive group (*P*<0.001) (Table 2). While the average amount of drainage was 450 ± 130 cc in Group I, it was 650 ± 120 cc in Group II. The mean drainage amounts were found to be significantly lower in Group I compared to Group II (*P*<0.001) (Table 2). While the need for erythrocyte suspension transfusion was 0.7 ± 0.8 in Group I, it was 1.8 ± 1.5 in Group II (*P*=0.012).

**Table 2:** Comparison of perioperative data.

	Group I (n=132) Mean ± SD (Median/Min-Max)	Group II (n=130) Mean ± SD (Median Min-Max)	*P*-value
Cross-clamping time (min.)	86 ± 13.2 (91/80-105)	62 ± 21.4 (69/59-94)	< 0,001^*^
Drainage amount (cc)	450 ± 130 (530/250-775)	650 ± 120 (720/370-905)	< 0,001^*^
ES transfusion (unit)	0.7 ± 0.8 (0/0-3)	1.8 ± 1.5 (1/0-6)	0,012^*^
Intensive care stay (day)	1.1 ± 0.4 (1/1-4)	2.0 ± 1.1 (2/1-7)	0,005^*^
Hospitalization (days)	5.2 ± 2.1 (6/5-8)	7.4 ± 3.2 (8/6-11)	0,004^*^
CPB (min.)	152.24 ± 36.4 (162/141-192)	102.24 ± 19.4 (106/92-131)	< 0,001^*^

* Statistically significant CPB=cardiopulmonary bypass; ES=erythrocyte suspension; SD=Standard deviation

**Fig. 3:**
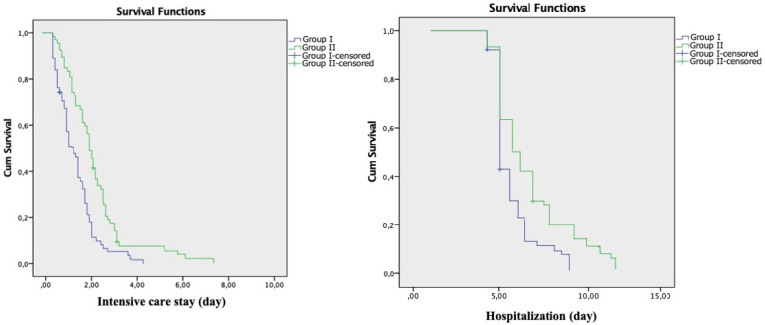
Analysis of intensive care and hospital stays with the Kaplan-Meier method.

The length of stay in the ICU of patients who underwent minimally invasive coronary revascularization was 1.1 ± 0.4 days; in the patient group who underwent conventional surgery, this period was 2.0 ± 1.1 days. These durations were found to be significantly shorter in Group I than in Group II (*P*=0.005). Kaplan-Meier analysis was performed and confirmed by the log-rank test (log-rank = 8.24; *P*<0.001) (Figure 3). The mean hospital stay of patients who underwent minimally invasive surgery was 5.2 ± 2.1 days; it was 7.4 ± 3.2 days in the patient group who underwent conventional surgery. This period was found to be significantly shorter in Group I than in Group II (*P*=0.004). Again, Kaplan-Meier analysis was performed, and the difference between them was confirmed by log-rank test (log-rank = 7.76; *P*=0.004) (Figure 3).

There were postoperative transient ischemic attacks without residual loss in two patients in Group I and in three patients in Group II. Six patients in Group I and seven patients in Group II were revised due to bleeding (Table 3). Postoperative renal dysfunction occurred in six (4.5%) patients in Group I and in eight (6.1%) patients in Group II. Pneumonia occurred in one (0.7%) patient in Group I and in two (1.5%) patients in Group II. Wound infection was observed in two (1.5%) patients in Group I and three (2.3%) patients in Group II. Pleural effusion was observed in 11 (8.3%) patients in Group I and nine (6.9%) patients in Group II. Adequate recovery was achieved with thoracentesis. Total perioperative mortality was one patient in both groups, and there was no significant difference in mortality (*P*=0.82).

**Table 3:** Comparison of postoperative complications.

	Group I (n=132)	Group II (n=130)	*P*-value
Transient ischemic attack	2 (1.5%)	3 (2.3%)	0.62
Postoperative arrhythmia	28 (21%)	32 (24%)	0.72
Postoperative renal dysfunction	6 (4.5%)	8(6.1%)	0.50
Wound infection	2 (1.5%)	3 (2.3%)	0.62
Reoperation for bleeding	6 (4.5%)	7 (5.3%)	0.71
Pleural effusion	11 (8.3%)	9 (6.9%)	0.65
Mortality	1 (0.7%)	1 (0.7%)	0.82

## DISCUSSION

Many modifications have been tried to increase the applicability of minimally invasive coronary surgery techniques. The fact that conventional sternotomy method approaches cause major trauma to the patient, delay in returning to daily life in the postoperative period, and cosmetic problems continue to direct surgeons to minimally invasive methods^[3]^. Considering all perioperative results, the quality and success of the surgical procedure must be high for minimally invasive methods to be applicable.

Minimally invasive CABG has been a safe alternative method to conventional CABG with sternotomy in experienced centers. Ruel et al.^[4]^ showed the graft patency statistics as 85% for saphenous vein grafts and 100% for LIMA in minimally invasive CABG methods performed by surgeons who have completed the learning curve in centers with sufficient experience. In 2009, McGinn et al.^[5]^ concluded that it can be safely applied in a prospective study of their dual-center experience with 450 patients undergoing minimally invasive CABG. Perioperative mortality rates were 1.3%. In another study, it was reported that complete revascularization was achieved in all 89 patients who underwent minimally invasive CABG. No perioperative mortality was observed^[6]^. Calafiore et al.^[7]^ published a large series extending the indication for minimally invasive CABG via left anterior thoracotomy to patients with multivessel coronary disease. Successful results in all these studies have contributed to the reliability of minimally invasive methods.

In the transition period to minimally invasive methods, video-assisted traditional thoracoscopic instruments were used, and development of robotic methods contributed to endoscopic techniques^[8^,^9]^. Taylor et al.^[10]^ emphasized the importance of the learning curve in minimally invasive cardiac surgery. Lima et al.^[11]^ conducted studies on thoracoscopic approaches in coronary artery surgery. In the following years, they competed with robot-assisted minimally invasive methods and direct approach. Diegeler et al.^[12]^ demonstrated that minimally invasive direct CABG is a safe procedure and can be performed safely in patients with multivessel coronary artery disease. In order to shorten the learning curve duration, Une et al.^[13]^ suggested in their study that the use of CPB in mini-thoracotomy for multivessel coronary revascularization contributes to surgical success and prevents the need for sternotomy conversion in patients at risk of intraoperative hemodynamic instability. By performing on-pump all of our minimally invasive coronary revascularization cases, we both increased the confidence in surgical quality and prevented negative results that may occur in the learning curve process. The use of CPB device has contributed significantly to the success of our case series. When positioning the heart for distal anastomoses, it must be evacuated by the pump so the heart can fold easily. With this method, it is possible to get the chance of anastomosis even in difficult areas of the heart.

In cases where CPB is used, the operation time may be longer compared to off-pump surgeries, but this prolongation is usually due to cannulation and decannulation procedures. We think this prolongation can be tolerated for surgical confidence and controlled with quality anastomosis. Although diagonal and LAD anastomoses are relatively easy, adequate and accurate manipulation of the heart is required for circumflex and posterior descending artery anastomoses. If the heart ventricles are filled with blood during these manipulations, a resistance is encountered. Therefore, vacuum-assisted venous drainage can be used when necessary to overcome this resistance in minimally invasive methods. Vacuum support significantly contributes to the evacuation of the heart and facilitates positioning. Another contribution will be jugular venous cannulation to reduce ventricular fullness.

Although the thoracotomy incision was made anterolaterally in many previous studies, we applied the anterior thoracotomy approach in our series. Since we make all proximal anastomoses on the aorta, the incision should be closer to the sternum in terms of easy access to the aorta. We did not perform any T or Y graft anastomosis on the LIMA graft. Anterior lateral thoracotomy will put more strain on surgeons, as the ascending aorta will remain further away from the surgical area. Therefore, we sutured all proximal anastomoses to the aorta with conventional methods. We were able to bring the aorta closer to ourselves by light traction with the help of the side clamp that we held the ascending aorta.

In this minimally invasive technique, patient evaluation with contrast-enhanced tomography is very important during the preoperative patient preparation process. Preoperative determination of the aorta and cardiac location of the patients may be important for the surgical strategy. Sometimes it may be necessary to make the thoracotomy incision through the third intercostal space in patients with a short ascending aorta. In patients with a lower-level heart or distal LAD lesion, it may sometimes be necessary to make an incision through the fifth intercostal space. In cases which it is not sure, it can be passed intraoperatively to an upper or lower intercostal space without any resection. In addition, it is possible to prevent complications caused by femoral cannulation with contrast-enhanced computed tomography imaging. Severe stenosis or calcifications in the iliac or terminal aorta are absolutely effects for determining our perfusion strategy.

Another disadvantage of the minimally invasive method may be post-thoracotomy pain, which patients may complain about after the operation. Therefore, intercostal and pectoral blockade can be applied before or during the operation so that the comfort of the patient is not affected too much in the postoperative period. It enables us to obtain satisfactory results for the patient in the postoperative period, and the disadvantage is eliminated compared to conventional methods.

When the minimally invasive method is compared with conventional methods, it may cause a longer surgical time at the beginning of the learning curve period. However, shorter times will be achieved as a result of the adoption of the procedure by the team and its frequent repetition. Since we completed the learning curve period in our own center, there is no longer a significant difference in surgical times between thoracotomy and sternotomy in our series.

### Limitations

This study has several limitations. First, as a retrospective, single-center analysis, our findings may be influenced by institutional practices, potentially limiting generalizability to other centers with varying levels of experience in minimally invasive coronary surgery. Second, although efforts were made to match patient groups, the lack of randomization may have introduced confounding variables that could impact outcomes. Finally, the learning curve associated with minimally invasive techniques may have influenced operative times and outcomes, and further studies with a larger cohort and multicenter participation are needed to validate our findings.

## CONCLUSION

The minimally invasive left anterior thoracotomy method can be performed safely and effectively with less blood loss and need for blood transfusion, shorter intensive care and hospital stays, and good cosmetic results.

The most important recommendation for surgeons is that they should spend the learning curve process in experienced centers, which will contribute to the successful spread of this method. Our results show that this method has superior features compared to conventional sternotomy and can be performed safely and successfully.
